# The Impact of Gelatin and Fish Collagen on Alginate Hydrogel Properties: A Comparative Study

**DOI:** 10.3390/gels10080491

**Published:** 2024-07-25

**Authors:** Adrianna Wierzbicka, Mateusz Bartniak, Joanna Waśko, Beata Kolesińska, Jacek Grabarczyk, Dorota Bociaga

**Affiliations:** 1 Institute of Materials Science and Engineering, Lodz University of Technology, 90-537 Lodz, Poland; adrianna.wierzbicka@dokt.p.lodz.pl (A.W.); mateusz.bartniak@dokt.p.lodz.pl (M.B.); jacek.grabarczyk@p.lodz.pl (J.G.); 2 Institute of Organic Chemistry, Lodz University of Technology, 90-543 Lodz, Poland; joanna.wasko@p.lodz.pl (J.W.); beata.kolesinska@p.lodz.pl (B.K.)

**Keywords:** fish collagen isolation, bio-ink, hydrogel, porcine gelatin, soft tissue engineering, 3D bioprinting, biomaterials

## Abstract

Hydrogel materials based on sodium alginate find versatile applications in regenerative medicine and tissue engineering due to their unique properties, such as biocompatibility and biodegradability, and the possibility of the customization of their mechanical properties, such as in terms of the individual requirements of separate clinical applications. These materials, however, have numerous limitations in the area of biological activity. In order to eliminate their limitations, sodium alginate is popularly applied in combination with added gelatin, which represents a product of collagen hydrolysis. Despite numerous beneficial biological properties, matrix materials based on gelatin have poor mechanical properties and are characterized by their ability for rapid degradation in an aqueous environment, particularly at the physiological temperature of the body, which significantly limits the independent application opportunities of this type of composition in the range of scaffolding production dedicated for tissue engineering. Collagen hydrogels, unlike gelatin, are characterized by higher bioactivity, dictated by a greater number of ligands that allow for cell adhesion, as well as better stability under physiological conditions. Fish-derived collagen provides a material that may be efficiently extracted without the risk of mammalian prion infection and can be used in all patients without religious restrictions. Considering the numerous advantages of collagen indicating its superiority over gelatin, within the framework of this study, the compositions of hydrogel materials based on sodium alginate and fish collagen in different concentrations were developed. Prepared hydrogel materials were compared with the properties of a typical composition of alginate with the addition of gelatin. The rheological, mechanical, and physicochemical properties of the developed polymer compositions were evaluated. The first trials of 3D printing by extrusion technique using the analyzed polymer solutions were also conducted. The results obtained indicate that replacing gelatin with fish collagen at an analogous concentration leads to obtaining materials with a lower swelling degree, better mechanical properties, higher stability, limited release kinetics of calcium ions cross-linking the alginate matrix, a slowed process of protein release under physiological conditions, and the possibility of extrusion 3D printing. The conducted analysis highlights that the optimization of the applied concentrations of fish collagen additives to composition based on sodium alginate creates the possibility of designing materials with appropriate mechanical and rheological properties and degradation kinetics adjusted to the requirements of specific applications, leading to the prospective opportunity to produce materials capable of mimicking the properties of relevant soft tissues. Thanks to its excellent bioactivity and lower-than-gelatin viscosity of the polymer solution, fish collagen also provides a prospective solution for applications in the field of 3D bioprinting.

## 1. Introduction

Hydrogels as polymeric materials have found their application in many scientific fields for over 60 years, with a particular focus on medicine. In this area, for the first time, the applied clinical hydrogel material was poly(2-hydroxyethylmethacrylate), which, thanks to the research work of O. Wichterle and D. Lím in the 1960s, was used to manufacture the first soft contact lenses [1,2,3]. The features distinguishing hydrogels from other materials are their high water content, plasticity, relatively low rigidity, and porosity, thanks to which they are similar to the tissues of the human body in terms of physical properties [2].

The hydrogel materials of natural origin are generally equipped with bioactive ligands creating an environment promoting cellular activity. They can be obtained by isolation from natural mammalian extracellular matrix (ECM) components or other sources [4]. Among the hydrogel materials of natural origin, one can distinguish, among others, alginate hydrogels, which may be obtained from brown algae. In 1970, the Food and Drugs Administration (FDA) approved alginate as a material popularly considered and safe capable of finding applications in food, pharma, and medicine [5]. Currently, alginate hydrogels are applied, among other applications, as biomaterials in drug delivery systems, tissue engineering and regeneration, 3D printing, and hydrogel dressings production. Their wide range of clinical applications is dictated by the fact that sodium alginate demonstrates high biocompatibility, low cytotoxicity, non-immunogenicity, and adjustable mechanical properties. In addition, it has the ability to reproduce natural tissue microenvironment and demonstrates high flexibility, which allows it to fill irregular cavities [5,6,7]. However, due to high hydrophilicity and lack of interactive domains, it does not itself provide an environment promoting adhesion and the proliferation of the eukaryotic cells [8]. Incorporating other biomaterials of natural origin into the structure of alginate, such as collagen or gelatin through the action of inherent functional groups improves adhesion and stimulates the proliferation of mammalian cells on the surface and inside of developed hydrogel materials [9].

Gelatin is formed as a product of the thermal denaturation of insoluble collagen, generally of bovine or porcine origin. It may be obtained only from sources rich in type I collagen, which does not contain cysteine [10]. Hydrogels based on gelatin are characterized by excellent biocompatibility and non-immunogenicity. Due to having RGD (arginine–glycine–aspartic acid) sequences in their structure, gelatin positively influences the cells’ adhesion, differentiation, and migration. Its characteristic feature is the ability for a thermo-reversible transition from sol into a gel, which occurs at a low temperatures of about 20–30 °C [7,11,12,13]. Gelation itself is the result of the binding of single polypeptide chains with the participation of hydrogen bonds. The gel strength increases proportionally to the increase in gelatin concentration and temperature drop [14]. Despite numerous beneficial biological properties, matrix materials based on gelatin have poor mechanical properties and are characterized by rapid dissolution and degradation in an aqueous environment, particularly at the physiological temperature of the body, which significantly limits the independent application opportunities of these types of compositions in the field of scaffoldings production dedicated to tissue engineering [12,15,16].

The most common occurring protein in the animal kingdom is collagen. It is a main component of the extracellular matrix. Depending on the distribution in individual tissues and the content of the triple-helical tertiary structure, at least 28 types of collagen are classified; however, of these in the human body, 90% constitute types I, II, and III collagen [17,18,19]. Collagen is a well-known material in tissue engineering due to its excellent biocompatibility and low immunogenicity. This polymer contains adhesive peptides and cell receptors, which promote cell attachment, proliferation, and migration. Collagen, similarly to gelatin, contains in its structure RGD sequences, but unlike gelatin, collagen hydrogels are characterized by higher bioactivity and a greater number of ligands which allow for cell adhesion as well as higher temperature stability. Unfortunately, the mechanical properties of collagen hydrogels are generally insufficient to be used on their own. Therefore, it is frequently combined with other biomaterials, such as alginate [12,19,20,21,22,23,24,25]. Due to the common occurrence of collagen in nature, the possibility exists of its isolation from serum, skin, bones, placenta, tendons, cartilage, and other tissues. In general, collagen is isolated through extraction from zoonotic material (cattle, pigs, rats, fish) or recombinant methods with the use of chemical hydrolysis methods using solubilization with acid, base, or salt, as well as by enzymatic hydrolysis with the application of proteolytic enzymes such as pepsin [17,26,27]. Currently, bovine spongiform encephalopathy, an epidemic of transmissible spongiform encephalopathy, and foot and mouth disease have caused severe limitations to the use and extraction of mammalian collagen. The possibility of bovine collagen being contaminated with prions was a consequence of the search for alternative protein sources, which are freshwater organisms, marine organisms, and mollusks [28,29,30,31]. Fish collagen has the advantage of religious neutrality, thanks to which this material may be used by Jews, Muslims, and Hindus, for whom both gelatin and collagen of bovine origin or porcine are prohibited [20,29,30,31,32,33,34]. These followers accounted for as much as 38.4% of the world’s total population in 2010 [20,31,35]. Due to its lack of religious and cultural restrictions, safety, low immunogenicity, lack of cytotoxicity, and extraction efficiency, fish-derived collagen has recently been the subject of a growing amount of research work. Fish collagen can also provide the basis for producing bioactive peptides, which can exhibit a higher ease of absorption antioxidant and anti-inflammatory effects [31,36,37,38]. Due to its lower imino acid content, however, fish-derived collagen is characterized by a lower thermostability and the ability to denature thermally at lower temperatures than mammalian-derived collagen [26,29,31].

Currently, broadly expanding techniques using common hydrogel materials are 3D bioprinting methods, representing the future of tissue engineering. These methods overcome the limitations of traditional tissue engineering techniques, which rely on the fabrication of scaffoldings and, subsequently, populating their surfaces with eukaryotic cells [39,40]. The conventional techniques applied so far generate numerous problems in the vascularization of newly formed tissues and the inhomogeneous distribution of biological material in the scaffolding structure [41]. The 3D bioprinting techniques allow for the generation of complex three-dimensional structures, the personalization of implants, and the fabrication of precise surface topographies [40]. Among 3D bioprinting methods, we can distinguish between direct and indirect printing techniques [42]. Direct bioprinting allows for the fabrication of the structures layer by layer using a computer-aided design (CAD) with the use of bioink compositions containing biological material in their structure [43]. Bioinks are, in general, produced with hydrogel materials of natural or synthetic origin. Due to excellent biocompatibility and bioactive properties among materials of natural origin in 3D bioprinting based on bioink extrusion, compositions based on alginate, hyaluronic acid, collagen, or gelatin, and created on their basis, hybrid materials are frequently used [42,43,44].

This study aimed to conduct a comparative analysis of the different compositions of hydrogel materials based on sodium alginate with such additives as gelatin or fish collagen in different concentrations. The research work carried out by our team to date has shown that hydrogel based on sodium alginate with added gelatin offers the most beneficial composition material in terms of the 3D bioprinting process of thin-walled structures and the viability and proliferation of eukaryotic cells incorporated in its structure [7,45]. Taking into account the numerous advantages of fish collagen, its better reflection of the natural extracellular matrix, excellent bioactivity of collagen peptides, and greater stability with respect to gelatin, the decision was made to analyze this component of hydrogel material compositions in terms of prospective application in tissue engineering and extrusion 3D printing processes.

## 2. Results and Discussion

### 2.1. Swelling Degree of the Hydrogel Materials

**Figure 1:**
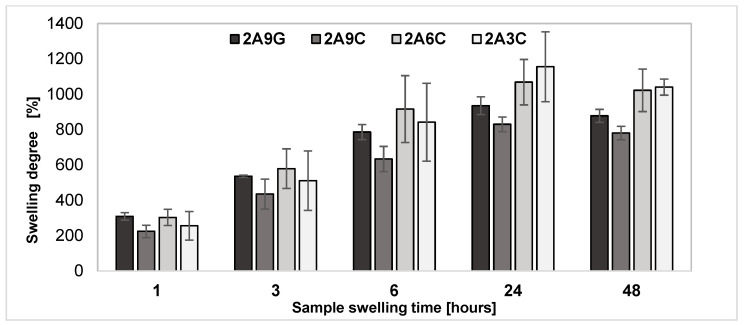
Evaluation results of the degree of swelling in the PBS buffer of the analyzed hydrogel materials at a temperature of 37 °C.

Swelling degree study enables the evaluation of the material’s ability for water or other fluids’ absorption, which is a key property for the potential applications of the material in clinical applications such as wound healing and tissue engineering. Swelling is a characteristic property of hydrogel materials based on sodium alginate, gelatin, and collagen. The results of the analysis of the degree of swelling of the prepared materials, shown in Figure 1, indicate that, over a period of 24 h, the samples showed a gradual increase in weight, which indicates that the swelling process is occurring for all the hydrogel compositions tested. Maximum swelling degree values for all types of the analyzed materials were observed after 24 h of incubation in PBS buffer. After 48 h incubation, the weight of all analyzed sample compositions begins to decrease, proving that the materials’ degradation process began.

After 24 h incubation, the highest swelling rate was observed for 2A3C samples. However, hydrogel samples containing 9% fish collagen demonstrate the lowest swelling degree among the analyzed materials. This observation is confirmed by the research conducted by the team of A. Sionkowska et al. The analysis of the degree of swelling of chitosan samples with different contents of fish collagen showed an analogous relation, indicating that the lowest degree of swelling was observed for materials with the highest concentration of collagen [46]. The results obtained also confirmed the research work conducted by the team of G. Montalbano et al., which indicates that higher collagen concentrations lead to the weakening of specific molecular interactions such as hydrogen bonding and dipole–dipole interactions. This phenomenon is directly associated with the protein hydration drop in the hydrogel. Therefore, with an increase in collagen concentration, a weakening of hydrogen bonds occurs, which affects the decrease in the degree of swelling of the material and limits the ability to retain water in the hydrogel network [47].

The analysis of the swelling process of materials with analogous concentrations of hydrogel components, i.e., samples 2A9G and 2A9C, showed that alginate hydrogels containing the added gelatin are characterized by higher swelling values in each case. The higher swelling degree values observed for the 2A9G material may be a result of gelatin’s lower molecular weight, its highly hydrophilic nature, and lower stability at an incubation temperature equal to 37 °C. All these factors may contribute to gelatin’s ability to absorb water and increase in volume, which is interpreted as swelling [12]. The results obtained confirm the study conducted by the team of J. Ratanavaraporn et al., which indicates that hydrogel scaffolds produced with 0.6% *w*/*v* collagen are characterized by lower porosity with respect to the scaffoldings of analogous gelatin concentration. However, the observed porosity and dense morphology of collagen-based materials allow collagen-based hydrogels to absorb fluids, which compensates for the hydrophobic properties of this material [48].

The observed differences in the swelling degree of alginate samples containing the addition of fish collagen or gelatin suggest that the type and concentration of hydrogel material additives significantly affect the hydrogel’s rate and swelling capacity.

The analysis carried out by Y. Li and T. Tanaka indicates that the gel swelling and shrinking processes are not pure diffusion processes. This phenomenon occurs due to the existence of the shear modulus of the network. The nature of the shear modulus is to maintain the shape of the system, which means minimizing non-isotropic deformation. The total energy of a gel can be divided into bulk energy and shear energy. The bulk energy of the system is related to the change in volume, which is controlled by diffusion. In contrast, the shear energy can be immediately minimized by readjusting the shape of the gel. Because there is no relative motion during the shear relaxation process and there is friction between the gel network and the solvent, the system can immediately adjust its shape to minimize the total shear energy. The effect of a non-zero shear modulus is to reduce the speed of the diffusion process. The reduction factor is directly related to the ratio of diffusion to overall dimensions. The theory predicts that the apparent collective diffusion constant and relaxation time are dependent on the geometry. The proposed model describes the combination of two processes: mass diffusion and mechanical relaxations of the polymer structure. It allows for accurate prediction of relaxation times and diffusion coefficients for hydrogel materials with different geometries, enabling a realistic description of gels’ swelling and shrinking dynamics. The collective diffusion coefficient can be determined by analyzing the experimental data on changes in hydrogel volume and matching them to a theoretical diffusion model [49].

### 2.2. Evaluation of Mechanical Properties of the Hydrogel Materials Using Static Compression Test

**Figure 2:**
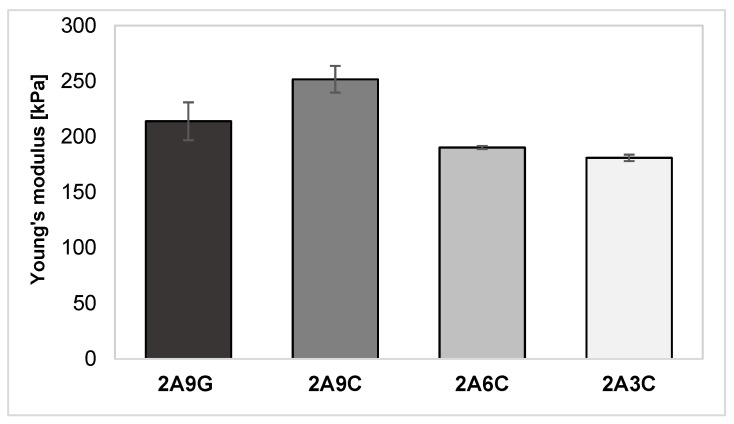
Results of the analysis of the mechanical properties of the developed hydrogel materials.

As shown in Figure 2, the mechanical properties evaluation test results of the prepared hydrogel materials indicate that the highest value of Young’s modulus is observed in hydrogel samples containing the highest concentration of fish collagen. The average value of Young’s modulus for this material amounts to 251.67 ± 12.07 kPa. The lowest average Young’s modulus value, equal to 180.94 ± 2.91 kPa, was observed for the material containing the lowest concentration of fish collagen among the samples analyzed. Young’s modulus is a measure of a sample’s resistance to elastic deformation under the influence of an applied force. Higher values of Young’s modulus indicate a more rigid and stronger material. The obtained results confirm, therefore, that the material’s rigidity increases with an increase in the applied concentrations of fish collagen [18,25]. It may also be noted that the most responsible for the mechanical strength of the developed hydrogel materials is sodium alginate cross-linked with divalent ions [50]. The research conducted by the team of E. O. Osidak et al. has indeed shown that Young’s modulus of Viscoll Type I collagen-based hydrogel materials with concentrations 20, 30, and 40 mg/mL amounts to accordingly 8.2 kPa, 9.5 kPa, and 21.5 kPa [51].

For samples containing equal concentrations of gelatin and fish collagen additives of 9%, the results of the static compression test indicate that the samples supplemented with the collagen addition are characterized by a higher value of Young’s modulus compared to the material containing an analogous concentration of gelatin, for which the value of longitudinal elastic modulus amounts to 213.87 ± 17.02 kPa. This observation indicates that using fish-derived collagen addition allows one to obtain materials with higher rigidity and consequently with better mechanical properties. This phenomenon is the result of the different structures of gelatin and collagen. The research of the team of J. Ratanavaraporn et al. indicates that, since the triple helix structure of collagen provides higher mechanical properties of the material, the gelatin, characterized by a randomly coiled structure, makes it possible to obtain a hydrogel material with lower mechanical strength [48].

A prospective direction of research work aimed at evaluating the mechanical properties of prepared hydrogel materials is the determination of shear modulus as a measure of material elasticity as a function of temperature at a particular hydrogel swelling degree and with different concentrations of protein additives in the form of gelatin and fish collagen [52]. An analysis of the shear modulus may have important consequences for the prospective interactions of the developed hydrogels with biological tissues and cells contained in 3D bioprinted structures by influencing the processes of cell adhesion, migration, and differentiation.

### 2.3. Evaluation of Changes in the Samples Weight after Individual Incubation Periods

**Figure 3:**
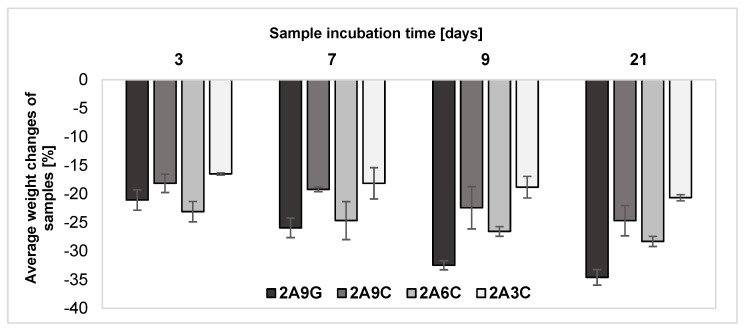
Results of the analysis of weight changes of the hydrogel samples during individual incubation processes.

The results of the analysis of weight changes of the hydrogel material samples during incubation processes carried out at a temperature of 37 °C in PBS buffer supplemented with antibiotics are shown in Figure 3. The results obtained from the weight changes of the samples after individual incubation periods indicate that each of the hydrogel materials underwent gradual degradation. After a three-day incubation process, the highest weight loss of 23.08 ± 1.78% was recorded for the material containing 2% alginate and 6% fish collagen, though a similar weight loss value equal to 21.05 ± 1.78% was also observed for samples containing gelatin addition. In subsequent incubation periods, the highest weight losses were registered each time for material supplemented with 9% gelatin addition. The lowest weight losses of each incubation period were observed for material containing the lowest concentration of fish collagen among those analyzed. After 21 days, weight loss for this material was 20.65 ± 0.54%. In comparison, after the same incubation period, the highest recorded weight loss was characteristic of the material with 9% gelatin addition—it amounted to 34.59 ± 1.36%. The obtained results also make it possible to note that material containing the lowest concentration of fish collagen is also characterized by the most stable degradation process over time. The higher weight loss obtained each time for the samples made from the 2A6C-based material compared to the 2A9C composition may result from the weakening of hydrogen bonds between water and gel components with increasing collagen concentration. This phenomenon significantly reduces the material’s ability to swell and retain water in the hydrogel network, as confirmed by the results described in Section 2.1. The reduced water retention potential of the hydrogel network results in its greater stability and limited ability to break the bonds of material components during degradation processes [47].

The lower degradation rate of 2A9C samples compared to materials containing a 9% concentration of gelatin is a result of the hydrophobic nature of collagen and its triple-helix structure, which, compared to hydrophilic gelatin, limits the swelling and solubility of the material, resulting in its slower degradation in physiological fluids [48]. This is because the complete degradation of collagen under physiological conditions occurs as a result of water and enzyme penetration and bond digestion. Due to the triple helix conformation, native collagen can be completely degraded under physiological pH and temperature conditions due to the action of enzymes that cleave the protein in the undenatured helical regions [10]. Noticeably, during the incubation periods discussed, the fastest degradation of 2A9G-based hydrogel materials occurs. This phenomenon can be correlated with the highest degree of swelling of the material containing the added gelatin. This is because hydrogel hydrolysis is accelerated by increased water absorption, which results in the breaking of covalent bonds and the rapid degradation of the material [25]. The fastest degradation of sodium alginate- and gelatin-based materials is also the result of the gelatin transition from the gel state to the solution at a temperature of 30–40 °C, which causes its gradual washing out of the material under physiological conditions. This property of thermally cross-linked gelatin limits its long-term use in transplantation [16,53].

During the degradation processes of sodium alginate-based hydrogel materials under physiological conditions, the exchange of divalent calcium ions cross-linking the alginate to monovalent ions such as sodium ions present in the degradation medium also occurs. This process leads to the gradual depletion of the network and decross-linking of the hydrogel, resulting in a slow dissolution of the material and an observed weight decrease in all analyzed sample compositions during the incubation processes [53,54].

### 2.4. Release of Calcium Ions from the Hydrogel Materials after Individual Incubation Periods

Calcium ion release is an important factor for hydrogel material applications in biomedicine and tissue engineering. This is because the calcium ions play a key role in several biological processes, such as blood clotting, nerve signal transmission, and muscle contraction. In the case of the potential applications of the developed hydrogel materials such as hydrogel bioinks, an excessively high a concentration of calcium ions may induce a cytotoxic effect against the cells placed inside the material [4,54].

**Figure 4:**
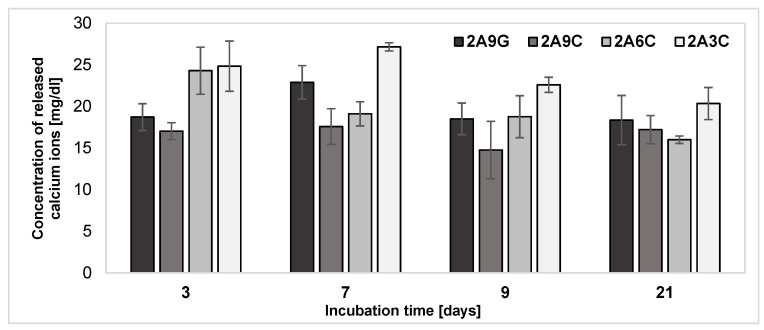
Results of the analysis of calcium ion release from the hydrogel samples during individual incubation processes.

The results of the analysis of calcium ion release from the developed hydrogel materials during individual incubation periods, as shown in Figure 4, indicate that, each time, the highest concentrations values of released calcium ions characterize the samples based on 2% alginate and 3% fish collagen. The lowest values of concentrations of released calcium ions are characterized by the composition containing the highest proportion of fish collagen in its structure. The only exception to this is the incubation time of about 21 days, during which a slightly lower concentration of released calcium ions is characterized by the composition enriched with 6% fish collagen. Therefore, we can conclude that the collagen concentration used to prepare the hydrogel may have a significant effect on the rate of calcium ion release. A higher concentration of collagen may result in a reduced rate of calcium ion release, while a lower concentration of collagen leads to a much more intense release of divalent ions cross-linking the hydrogel. Literature data, being the result of the research work of the team of Y. Chen et al., indicate that the shrinkage rate after cross-linking materials containing sodium alginate and collagen increases as the collagen concentration increases. This phenomenon is the result of the high mobility of free calcium ions towards their formation of complexes with -COOH groups. When low concentrations of collagen are used, a rapid reaction of calcium ions with sodium alginate occurs, which limits the penetration of the fiber. More uniform cross-linking occurs when calcium ions penetrate the fiber, which is possible for higher concentrations of collagen [53,54,55]. The process of the partial decross-linking of the material occurs through the release of calcium ions from the analyzed materials, and therefore occurs more intensively for samples with the least uniform cross-linking, where there is no internal fibers’ penetration.

A comparative analysis of the concentrations of released calcium ions from samples containing 9% gelatin and fish collagen additives demonstrated that, during all incubation processes, the release of higher concentrations of calcium ions is observed from the samples containing the added gelatin in their structure. This phenomenon may be due to the hydrophilic nature of gelatin, described in previous sections, the higher porosity of the hydrogels it forms, and its ability to take in a liquid form at the incubation temperature of the samples [12,16,48,53]. The faster washout of protein from the alginate matrix may, therefore, cause an intensified release of calcium ions cross-linking the hydrogel. So, the application of fish collagen in appropriate concentrations as an addition to sodium alginate-based hydrogel materials can limit the level of calcium ions released into the degradation environment. These observations also provide confirmation of the results of the analysis of weight changes in hydrogel printouts discussed in Section 2.3. Since the process of calcium ion release correlates with the gradual decross-linking of the alginate hydrogel due to ion exchange, this increases the material degradation rate. The dissolution of alginate hydrogels in PBS buffer results from the long-term ineffectiveness of the physical cross-linking of chains with Ca^2+^ ions. Since it has been shown that, when intramolecularly cross-linked with divalent calcium cations, guluronate blocks of alginate chains tend to undergo ion exchange between sodium and calcium in a physiological environment, leading to a loss of their mechanical properties and elasticity [53,54,55,56]. This characteristic is a desirable phenomenon if, during the processes of ion exchange and material degradation due to cellular activity, a gradual replacement of the ECM implant occurs [4].

The analysis of the release of calcium ions from hydrogel materials based on sodium alginate and collagen indicates that the change in mass of the samples is not a process with the same dynamics as the decross-linking of alginate due to ion exchange. This thesis is confirmed by the results achieved for the 2A3C composition, for which the lowest degree of hydrogel mass loss was recorded each time during incubation periods of 3, 7, 9, and 21 days. In contrast, the results of calcium ion release achieved for the same composition indicate that these samples are characterized by the lowest concentrations of released Ca^2+^ ions cross-linking the alginate matrix during degradation. Changes in the mass of the analyzed materials are dynamic processes in which the recorded values increase linearly with time. In contrast, the processes of calcium ion release from hydrogel samples are less dynamic. For all analyzed materials, the most intense release of calcium ions occurs after 3 and 7 days of degradation. This phenomenon may be the result of an initially dynamic exchange between the calcium ions cross-linking the alginate matrix and the sodium ions available in the degradation medium of the samples. The kinetics of calcium ion release from hydrogel materials stabilizes when the system becomes progressively saturated. It is also important to highlight that an intensive process affecting the values of the degree of mass change of the developed materials over time is the release of proteins contained in the hydrogel matrices.

### 2.5. Release of Protein from the Hydrogel Materials after Individual Incubation Periods

**Figure 5:**
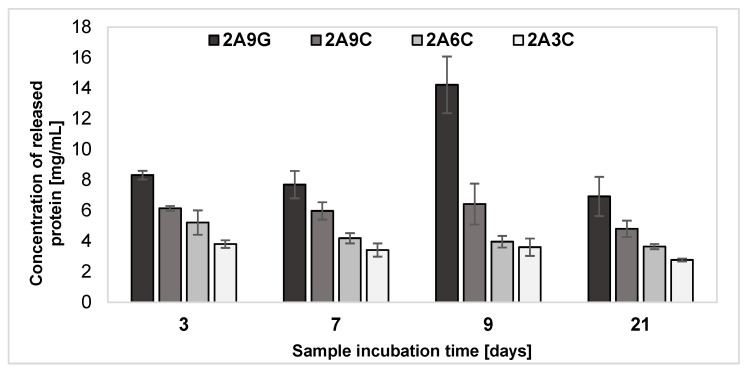
Results of the analysis of protein release from the hydrogel samples during individual incubation processes.

The results of the analysis performed by the Lowry method of the release of proteins from the developed hydrogel materials during their incubation processes for individual periods, shown in Figure 5, indicate that the material enriched with the addition of gelatin each time demonstrates the highest concentration of released proteins into the degradation medium among the analyzed systems. This phenomenon is dictated by the nature of chemically non-cross-linked gelatin, described in Section 2.3, which, at the physiological temperature, begins to form a liquid by thermo-reversible cross-linking and undergoes gradual dissociation [53]. Under such conditions, the gelatin not bound to the cross-linked blocks of alginate undergoes liquefaction, which in turn leads to its gradual washing out from the material. Collagen-based hydrogels demonstrate the gradual release of proteins over time. Among the samples with collagen addition, the lowest concentrations of proteins released into the degradation medium were observed for the material containing the lowest equal to 3% concentration of collagen. At the same time, the highest concentrations of released proteins were recorded each time for materials supplemented with the addition of 9% fish collagen. Thus, it can be concluded that the amount of released proteins from the samples containing the collagen additive increases with the original protein concentration in the hydrogel compositions.

The maximum concentration of proteins released during degradation processes by hydrogel samples with the 2A9G composition during 9-day incubation may be the result of the overall diffusion of gelatin from the hydrogel matrix during degradation processes occurring at increased temperature, in which gelatin liquefies and diffuses into the incubation environment of the samples. Literature data indicate that calcium ions released from the alginate matrix during the incubation process can selectively combine with the carboxyl groups of amino acids contained in the gelatin polypeptide backbone. These segments are very flexible, which simplifies the bonding —COO^−^—Ca^2+^—^−^OOC—between adjacent chains to form complexes. Under these conditions, a gel is formed, which represents a hybrid structure. This observation indicates that such larger clusters can be formed in gels containing Ca^2+^ ions, which are not part of the network and are able to diffuse into the medium. The size of these clusters increases with salt concentration [57]. The presence of additional complexes in the incubation medium of the samples can block the detection of free proteins in the degradation medium and affect the achieved reduced protein concentration after 21 days, disturbing the obtained Lowry method measurement results. In fact, proteins forming complexes are characterized by a changed structure, which may affect the availability of amino groups necessary for reacting with the Folin–Ciocalteu reagent.

Diffusion can occur in two distinct areas—separated by a moving boundary or interfacial surface. In hydrogel materials, the boundary of the diffusion region changes its position over time. As the degradation medium diffuses into the hydrogel, sharp boundaries are observed, corresponding to discontinuities in the concentration–distance curve gradient. During the degradation, the external boundary of the hydrogel changes its position. The boundary’s movement depends on the material’s degradation rate under the environmental conditions. The moving boundary can be described in terms of the change in volume and mass of the hydrogel that occurs during degradation. The mathematical modeling of this phenomenon can be carried out using Josef Stefan equations, which take into consideration the diffusion equation, the edge conditions on the moving boundary, and the initial conditions. The application of diffusion theory with a moving boundary can enable a comprehensive understanding of controlled release processes from degradable hydrogel materials [58].

### 2.6. Analysis of pH Changes of the Degradation Medium

**Figure 6:**
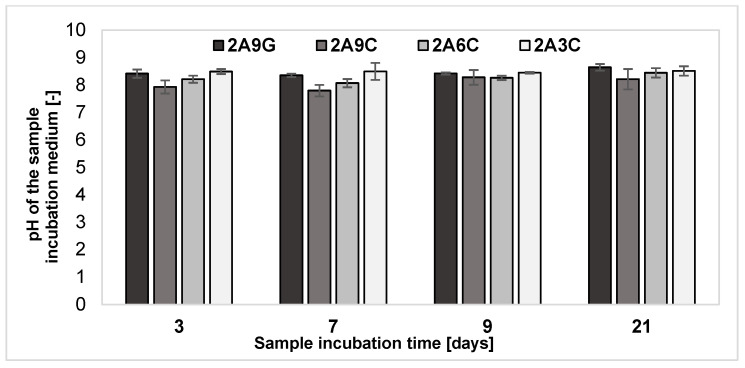
Results of the analysis of pH changes in the incubation medium of the hydrogel samples during degradation processes.

The results of the analysis of changes in the pH of the incubation medium of hydrogel samples, presented in Figure 6, showed that, for all the tested material compositions, the pH of the degradation environment of the samples remains at a similar level equal to about 8. Slightly lower pH values of the incubation medium were registered for the material containing 2% sodium alginate and 9% fish collagen. These data may be the result of the gradual release of proteins into the degradation medium of the samples, as confirmed by the results described in Section 2.5.

It should be noted that the pH of the incubation environment of hydrogel materials and polymer solutions may significantly affect the properties of the materials, including stability, degradation rate, and protein release. The strong monitoring and control of pH during the various stages of hydrogel material preparation and their incubation is essential to ensure and maintain the constant and desired properties of the hydrogel.

### 2.7. Evaluation of Rheological Properties of the Polymer Solutions

**Figure 7:**
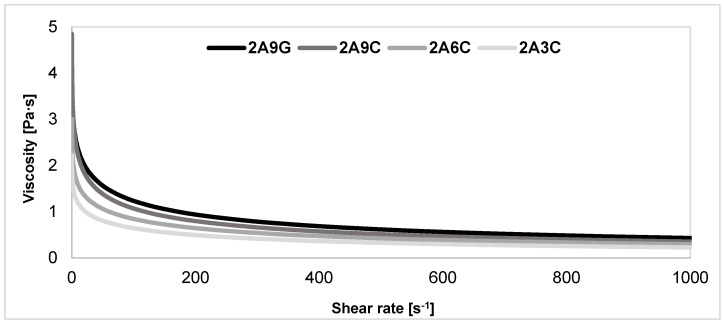
Results of the analysis of rheological properties of the developed polymer solutions. The results are an average of three measurements for each polymer solution composition.

The results of the analysis of the rheological properties of the developed polymer solutions, shown in Figure 7, indicate that all the materials are characterized by the ability to thin under the action of shear forces and are non-Newtonian liquids. The ability to thin as a result of increasing the shear rate suggests that the viscosity of the material decreases and consequently makes it easier to process under the action of shear forces. This property proves that these materials are suitable for applications where the polymer solution must be extruded through the nozzle without clogging it, being injected, or distributed [25,59,60]. The highest viscosity characterizes the composition containing 9% of gelatin addition. A slightly lower viscosity was registered for a solution with 9% fish collagen content. The lowest viscosity was registered for the 2A3C solution. Therefore, we may note that, as the concentration of collagen increases in hydrogel bioink compositions with equal sodium alginate content, the viscosity of polymer solutions increases [59]. The non-Newtonian liquid characteristics observed in solutions containing alginate and gelatin, or fish collagen, is appropriate for hydrogel and biopolymer solutions. It results from the complex network structure of these materials. Registered differences in viscosity between solutions containing gelatin and fish collagen indicate the potential of fish collagen as an alternative to gelatin in 3D bioprinting applications, as it exhibits similar rheological properties but at a lower polymer solution viscosity.

### 2.8. Three-Dimensional Printability of Prepared Polymer Compositions

Presented in Table 1, the results of the analysis of the printability of the developed composition polymer solution demonstrate that each prepared material based on alginate may be successfully applied in the extrusion 3D printing technique. The developed hydrogel materials compositions are characterized by a good pneumatic extrusion capability. Each of the 3D-printed structures with different hydrogel compositions, after cross-linking them using 1% calcium chloride solution, is characterized by a reproducible printed structure with a constant wall thickness and a preset non-folding tubular geometry. Previous research work conducted by our team has pointed to the use of a hydrogel composition based on 2% sodium alginate and 9% gelatin as the most optimal from the point of view of 3D bioprinting applications. The 3D-printing trials carried out within this study’s framework show that, thanks to its suitable rheological properties described in Section 2.7, fish collagen provides a prospective alternative hydrogel material that can be used in the 3D printing area.

Depending on the concentration of fish collagen addition used for hydrogel materials based on 2% sodium alginate, the extrusion pressure parameters vary during the 3D printing process. Together with an increase in the concentration of fish collagen in the polymer solution under consideration, it is necessary to apply a slightly higher extrusion pressure, which is correlated with the increasing viscosity of materials with a higher protein content. The research work of the team of Y. Chen et al. confirm that, using different concentrations of collagen in sodium alginate-based hydrogel materials causes a change in the rheological properties of polymer solutions, and, thus, directly affects the printability of the compositions analyzed. Improved printability, shape fidelity, and reduction in printout deformation are possible when using polymer solutions with higher concentrations of component application [59].

The developed tubular geometry of the printouts is beneficial for prospective 3D bioprinting processes. The thin-walled structure allows for the even diffusion of nutrients into cells embedded inside the material. Indeed, literature data show that a capillary diffusion limit is observed in uninoculated hydrogel scaffolds, which means that the biological material located in the core of the multilayer printout is supplied with limited amounts of nutrients and oxygen [60]. The use of a tubular printout geometry, therefore, allows for uniform flow of the culture medium between the walls of the printout, allowing for the even access and diffusion of nutrients to the biological material throughout the construct.

## 3. Conclusions

A comparative analysis of the properties of hydrogel materials based on sodium alginate with the addition of gelatin or fish collagen carried out within the framework of this study indicates that the use of collagen may serve as a prospective solution in the area of tissue engineering and 3D printing. It was demonstrated that the type and concentration of protein additives to the alginate matrix significantly affect its rheological properties, swelling capacity, mechanical properties, as well as hydrolytic degradation processes, and the 3D printing capabilities of the developed polymer compositions. The studies carried out within the framework of this work indicate that the replacement of gelatin with fish collagen at an analogous concentration leads to obtaining materials with a lower degree of swelling, better mechanical properties, and slowed degradation processes, consisting of the reduced kinetics of the release of calcium ions cross-linking the alginate matrix and limited protein release processes compared to gelatin, which are the result of the higher stability of collagen under physiological conditions. Thanks to its excellent bioactivity, a higher amount of ligands stimulates cell adhesion, as well as better stability under physiological conditions, and fish collagen also provides an alternative to gelatin in 3D bioprinting applications, as it exhibits analogous rheological properties with lower polymer solution viscosity. The selection of the appropriate concentrations of fish collagen additives to sodium alginate-based hydrogel compositions can make it possible to obtain materials capable of mimicking the properties of the body’s relevant soft tissues. In addition, the materials enriched with higher concentrations of fish collagen may offer the potential for applications in the area of tissue engineering, in which a hydrogel with higher rigidity and better mechanical properties is required. This work, therefore, serves as an introduction to optimizing the composition of hydrogel compositions based on sodium alginate and fish collagen in terms of the applications in direct 3D bioprinting processes.

## 4. Materials and Methods

### 4.1. Materials

A hydrogel material composition based on the sodium salts of alginic acid from brown algae (Sigma Aldrich, USA), gelatin from porcine skin type B (Sigma Aldrich, Burlington, MA, USA), and fish-derived collagen in lyophilized form were used for this study. Hydrogel solutions were prepared in a DMEM culture medium (Biowest, Nuaillé, France). Before the preparation process of hydrogel solutions, appropriate amounts of sodium alginate and gelatin powders underwent UV radiation sterilization for 1 h. The lyophilized fish collagen was not subjected to sterilization processes. A 1% solution of calcium chloride (Sigma Aldrich, USA) prepared in saline was used to cross-link the separate polymer composition. After the cross-linking process, the samples were rinsed with PBS buffer (phosphate-buffered saline) (Capricorn Scientific GmbH, Ebsdorfergrund, Germany). During the degradation processes of the developed materials, the PBS buffer supplemented with 1% Amphotericin B (Corning, New York City, NY, USA) and 1% penicillin–streptomycin–neomycin (P/S/N) (Sigma Aldrich, USA) was used.

### 4.2. Fish Collagen—Isolation and Obtaining the Test Material

The skins of the silver carp (*Hypophthalmichthys molitrix*) were used to obtain the collagen. In the first stage, the skins were cleaned of remaining scales, fins, and gills, thus preparing the cell layer for the further elimination of undamaged morphotic elements. Next, rinsing with a stream of fresh, clean water was performed until odorless, clear, biologically and chemically clean water was obtained. The next stage consisted of removing the remaining impurities, after the mechanical processing, from the cells located in the connective tissue substrate. Further procedures involved repeating the rinsing, which made it possible to obtain a raw material in which the secondary and tertiary order structures of the fibrous proteins are preserved. The next stage in obtaining the raw material involved rinsing the skins in hydrogen peroxide solution with a concentration of 2%. Prepared in the above manner, the raw material was used for further processing. The next element involved the hydration process leading to the collagen gel being obtained. A 1.6% lactic acid solution was used. The last part of the process involved filtration on cascade filters (filter gradations gradually decreasing from 400 µm to 20 µm). Filtration allows for the proper purification of collagen, and obtaining the highest possible density and viscosity. The obtained collagen gel was characterized by a pH of 3.8 and a denaturation temperature of 29.0 °C. Finally, the collagen gel was subjected to lyophilization (lyophilization temperature −48 °C).

The FTIR method was used to confirm the presence of the helical structure of collagen. In addition, the material’s morphology was determined using the SEM technique.

Infrared spectroscopy (FTIR) can be used to identify the compounds and groups of compounds that build given molecules. In special cases, it can be used to analyze molecular interactions, which have a significant impact on the formation of supramolecular structures. FTIR spectra were performed using an FTIR spectrometer VERTEX 70 (Bruker, Bremen, Germany) using an ATR Golden Gate adapter. A resolution of 2 cm^−1^ was used with 64 scans in the 600–4000 cm^−1^ range. The results were recorded in the program BRUKER OPUS 6.5 (Version 6.5, Bruker, Kennewick, WA, USA). Based on FTIR analysis of the sample, it can be concluded that the protein material studied has a triple helix structure. In the sample studied, amide I bands are observed in the range of 1650–1660 cm^−1^, amide II bands in the range of 1550–1560 cm^−1^, and amide III bands in the range of 1240–1250 cm^−1^, and bands corresponding to the stretching vibrations of NH groups in the range of 3330–3400 cm^−1^ are observed. The typical range of stretching vibrations for NH groups in proteins is the range of 3400–3440 cm^−1^. This band shifts towards lower frequencies when NH groups are involved in the formation of hydrogen bonds in a protein or peptide.

**Figure 8:**
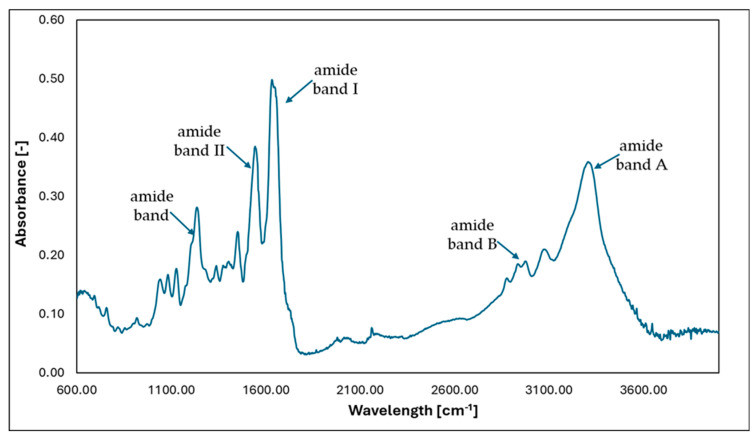
FTIR spectra of the sample of the collagen lyophilizate.

The spectra shown below (Figure 8) indicate that, in all cases, collagen has characteristic bands:

- Amide band A—3000–4000 cm^−1^—stretching vibrations from NH and NH_2_ groups;
- Amide band B—2880–2980 cm^−1^—asymmetric vibrations of CH_2_ groups;
- Amide band I—1600–1750 cm^−1^—stretching vibrations from amide C=O groups;
- Amide band II—1500–1600 cm^−1^—stretching vibrations of NH groups originating from CN groups;
- Amide band—1230–1245 cm^−1^—stretching vibrations of NH groups originating from CN groups.

**Figure 9:**
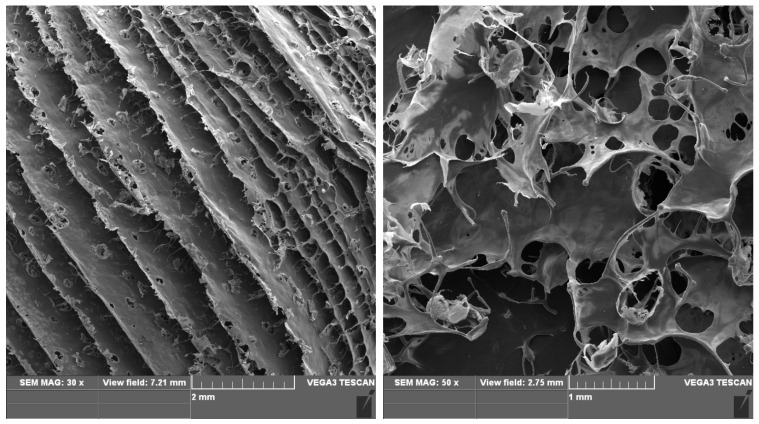
SEM images of the lyophilizate of the collagen material. Microscopic analysis of the individual samples was performed three times.

SEM images of a sample of the collagen lyophilizate (Figure 9). The tests were performed on a VEGA3 TESCAN (Tescan Osay Holding, Brno, Czech Republic).

The lyophilized collagen was also subjected to CD testing. CD spectra were registered using a Jasco J-1500 camera. Measurements were made in a quartz cuvette with an optical path length of 1 mm, resolution of 5 nm, slit widths of 4 nm, and a scanning speed of 100 nm/min. The test was carried out for collagen samples dissolved in water with HPLC purity. In addition, the tests were performed for collagen samples dissolved in water of HPLC purity with an added 5% by weight of lactic acid compared to the collagen used. The tests were conducted at a room temperature of about 25 °C.

**Figure 10:**
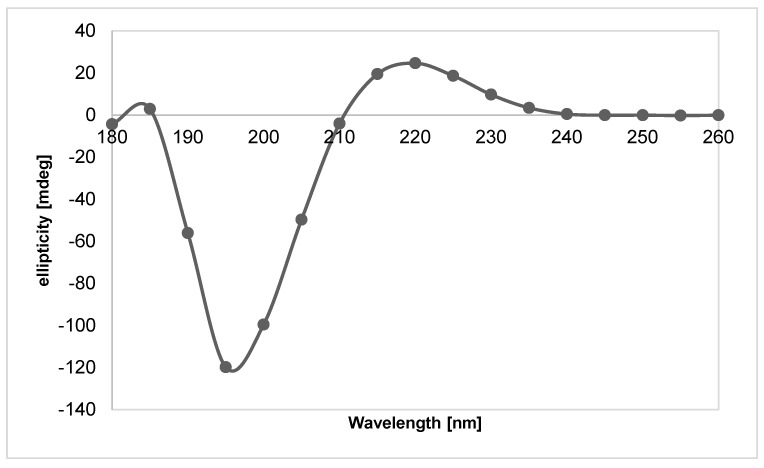
CD spectrum of the obtained collagen. The results are an average of three measurements.

**Figure 11:**
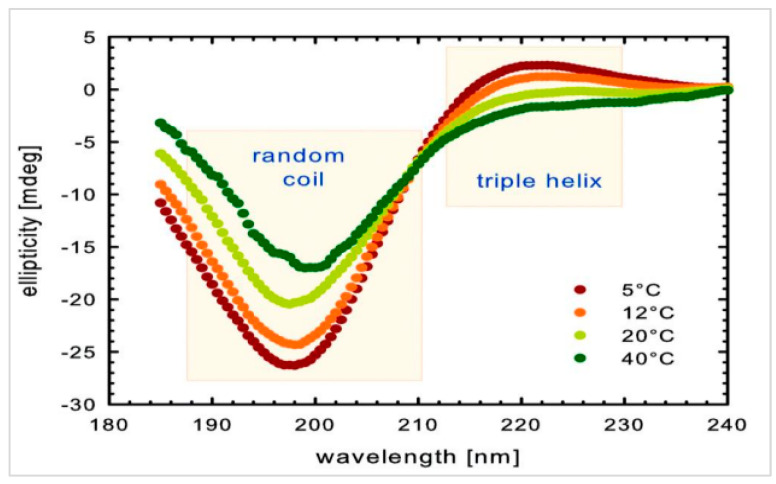
Model CD spectra of the collagen as a function of temperature. The results are an average of three measurements.

Based on the CD studies (Figure 10 and Figure 11), it can be concluded that the applied isolation and lyophilization process of collagen does not disrupt the protein’s I and III order structure, as the maximum that is characteristic of the triple helix of collagen is visible.

### 4.3. Preparation of the Hydrogel Materials

**Figure 12:**
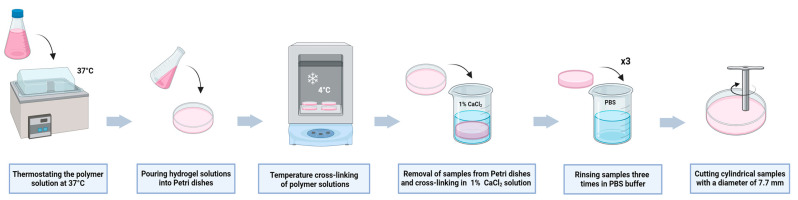
Schematics of the preparation process of the analyzed hydrogel samples. Source: elaborated by authors using BioRender.com [61].

Four 2% (*w*/*v*) sodium alginate solutions were dissolved separately in DMEM culture medium using a magnetic stirrer for 1 h at a temperature of 37 °C at a speed of 150 RPM. Appropriate additives were added to the sodium alginate solutions in the form of gelatin or fish collagen in weight/volume ratios with the aim of obtaining the final concentrations of the solution given in Table 2. The finished compositions were stirred on a magnetic stirrer for 1 h at a temperature of 40 °C at a speed of 150 RPM. The prepared polymer compositions were placed in a water bath at a temperature of 37 °C for 10 min with the purpose of obtaining a uniform material temperature across the entire volume. Polymer solutions were poured onto the surface of Petri dishes to form samples of identical height. The poured samples were pre-cross-linked at a temperature of 4 °C for 15 min to allow the hydrogel to separate from the Petri dish’s surface. The compositions were then cross-linked using a 1% solution of calcium chloride for 70 min. The cross-linking process was performed at room temperature. After the cross-linking process, each sample was rinsed in PBS buffer three times. The fabricated structures of each hydrogel material were then divided into smaller cylindrical samples with identical dimensions—5 mm high and 7.7 mm in diameter—using the ring of stainless steel. The process of the preparation of hydrogel samples is shown in Figure 12.

### 4.4. Swelling Degree of the Hydrogel Materials

The prepared samples of individual hydrogel materials were weighed and dried after fabrication at a temperature of 37 °C to a constant weight. The dry hydrogel was then subjected to swelling degree analysis to assess its ability to absorb and retain fluid. The swelling process was carried out in buffer PBS at a temperature of 37 °C for 48 h. At specified periods of time equal to 1, 3, 6, 24, and 48 h, the samples were taken out of the solution, dried off the excess fluids on a dust-free paper, and weighed again. The swelling tests were carried out for the five samples of each analyzed hydrogel material. Based on the weight change of the samples after each separate period of the incubation, the average swelling degree was determined using the following relationship (1):

(1) $$Swelling\;degree\left[\%\right]=\frac{W_{1}-W_{2}}{W_{2}}\times 100\%$$

where: *W*_1_—the weight of the hydrogel in the swollen state; and W_2_—the dry weight of the hydrogel.

### 4.5. Evaluation of Mechanical Properties of the Hydrogel Materials Using Static Compression Test

Evaluation of the mechanical properties of the prepared hydrogel materials was carried out using a static compression test with a BRUKER tribotester (UMT-2 Bruker, Billerica, USA) at a constant compression speed of 0.1 mm/s. The Young’s modulus values of the developed materials were determined based on the initial linear range of the stress–strain curve. Measurements were made for five samples of each of the hydrogel materials analyzed.

### 4.6. Preparation of the Extracts

The different samples of hydrogel materials were subjected to incubation processes in PBS buffer supplemented with 1% Amphotericin B (Corning, USA) and 1% P/S/N (Sigma Aldrich, USA) for periods equal to 3, 7, 9, and 21 days at a temperature of 37 °C. Extracts were prepared according to ISO10993-5 using the relationship: 1 mL of solution per 0.2 g of sample [62]. Three individual samples of each hydrogel material underwent analysis for each independent incubation period. The reference extract was a PBS buffer supplemented with Amphotericin B and P/S/N. After each incubation period, the samples were weighed to evaluate their weight changes during degradation processes. The extracts obtained were also used to determine the degree of release of calcium ions and proteins during incubation processes and changes in the pH of the degradation environment of the samples.

### 4.7. Evaluation of Changes in the Samples Weight after Individual Incubation Periods

Before the process of incubation, the samples of hydrogel materials were weighed. They were then subjected to individual incubation periods of 3, 7, 9, and 21 days at a temperature of 37 °C, according to Section 4.6. After each incubation period, the samples were removed from the degradation medium, dried on dust-free paper to remove excess liquid, and weighed again. Based on the results obtained, using the below relationship (2), the average percentage change in the weight of the samples after their individual incubation periods was determined:

(2) $$Change\;in\;sample\;weight\;\left[\%\right]=\frac{W_{1}-W_{0}\;\;\;\;}{W_{0}\;\;}\times 100\%$$

where: *W*_0_—weight of the sample before the incubation process; *W*_1_—weight of the sample after incubation process.

### 4.8. Release of Calcium Ions from the Hydrogel Materials after Individual Incubation Periods

The release of calcium ions used for cross-linking sodium alginate-based hydrogel materials was analyzed using extracts obtained according to the procedure described in Section 4.6. The study was conducted to evaluate the concentrations of calcium ions released into the surrounding degradation medium after the individual incubation periods of the samples. The concentration of calcium ions released into the degradation medium of the samples was analyzed by a spectrophotometric method using the Calcium CPC kit (BIOMAXIMA, Lublin, Poland). Absorbance measurements were made using a PERKIN ELMER VICTOR X4 spectrophotometer (PerkinElmer, Inc., Shelton, CT, USA) at 570 nm wave number.

### 4.9. Release of Protein from the Hydrogel Materials after Individual Incubation Periods

The study of the release of proteins from the samples of the analyzed hydrogel materials into the degradation medium of the samples was carried out using Lowry’s colorimetric method. Bovine albumin (BSA) was used as a serum reference protein. The extracts were analyzed with the use UV–Vis spectrophotometer (Ultrospec 21000 Pro, Biochrom Ltd., Cambridge, UK) with absorbance measuring at 750 nm. The results obtained were interpreted in terms of gelatin and collagen release from the hydrogel samples during individual incubation periods.

### 4.10. Analysis of Changes in the pH of Degradation Medium

The extracts of the hydrogel samples obtained after the separate incubation periods of the structures were used to analyze the degradation medium’s pH changes. The solution’s pH tests were performed using a pH meter of Mettler Toledo Sevencompact Duo S213 (Mettler Toledo, Columbus, OH, USA) at room temperature.

### 4.11. Evaluation of Rheological Properties of the Polymer Solutions

A rheometer of MCR702 Multidrive (Anton Paar GmbH, North Ryde, Australia) was used to examine the rheological properties of the analyzed polymer compositions. The polymer solutions were placed on the lower measurement plate of the device, and a gap between the measuring plates was set to 0.5 mm, using the geometry of a 25 mm diameter parallel plate. The measurements were carried out at a temperature of 34 °C, which is analogous to the typical conditions of the 3D printing of hydrogel materials based on sodium alginate and gelatin. The viscosity was measured in the shear rate speed range of 0.1–1000 s^−1^. Before each measurement, the polymer solutions underwent thermostating for 10 min with the purpose of obtaining an even polymer solution temperature distribution over the volume of the material.

### 4.12. Three-Dimensional Printing Process

**Figure 13:**
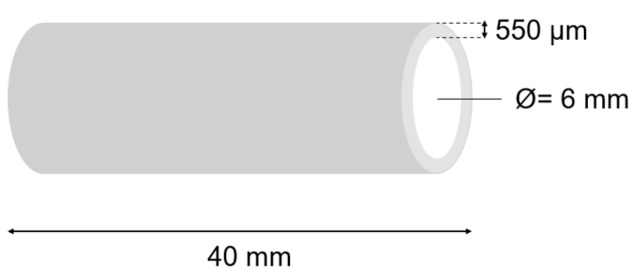
Three-dimensional printing schematics.

The individual polymer solutions were placed in separate, sterile syringes with a capacity of 5 mL. These compositions were used for the 3D direct printing of tubular structures with a length of 40 mm and an inner diameter of 6 mm (Figure 13). The 3D printing process was performed using a 3D printer that was designed and constructed at the Technical University of Lodz. The 3D printing process, during which the material is pneumatically extruded, was performed on a horizontal shaft using a straight nozzle with a diameter of 330 µm. The height of the print layer was set at 550 µm. The head movement speed was 0.23 mm/s, and the shaft rotation speed was equal to 46 RPM. The 3D printing process was conducted at a temperature of 34 °C. The extrusion pressure varied depending on the composition of individual polymer solutions (accordingly for 2A3C—16 kPa, 2A6C—18 kPa, and 2A9—21 kPa, respectively). The printed tubular structures were cross-linked on the shaft using a 1% CaCl2 solution. The cross-linking time of each of the samples was 10 min. The cross-linking process was performed at room temperature.

The printability of the material was assessed in two ways:

1. The ability of the hydrogel to flow through the printer nozzle without clogging or excessive spillage of the material was tested. Tests for this feature include flow analysis at various pressures and printing speeds.
2. The behavior of the material was assessed and analyzed immediately after the printing process, whether it maintained its required printed structure or flowed and dripped from the working roller.

If the material extrusion was possible, and the required structure could be formed from it without uncontrolled material spillage, it was considered printable using a given device, with the presented printing parameters that were considered optimal for a given hydrogel.

## Figures and Tables

**Table 1:** Results of the printability test of the analyzed compositions of hydrogel materials.

The Analyzed Hydrogel Composition	2A9G	2A9C	2A6C	2A3C
**3D Printability**	Possibility of 3D printing	Possibility of 3D printing	Possibility of 3D printing	Possibility of 3D printing

**Table 2:** Summary of hydrogel compositions analyzed during the study.

Symbol of the Hydrogel Composition	Concentration % (*w*/*v*)		
	Sodium Alginate (A)	Gelatin (G)	Fish Collagen (C)
**2A3C**	2	-	3
**2A6C**	2	-	6
**2A9C**	2	-	9
**2A9G**	2	9	-

## Data Availability

The data that support the findings of the current study are listed within the article.

## References

[1] Yang J.M., Olanrele O.S., Zhang X., Hsu C.C., Chun H., Park K., Kim C.H., Khang G. (2018). Fabrication of Hydrogel Materials for Biomedical Applications. Novel Biomaterials for Regenerative Medicine.

[2] Caló E., Khutoryanskiy V.V. (2015). Biomedical applications of hydrogels: A review of patents and commercial products. Eur. Polym. J..

[3] Wheeler J.C., Woods J.A., Cox M.J., Cantrell R.W., Watkins F.H., Edlich R.F. (1996). Evolution of hydrogel polymers as contact lenses, surface coatings, dressings, and drug delivery systems. J. Long. Term. Eff. Med. Implant..

[4] Di Giuseppe M., Law N., Webb B., Macrae R.A., Liew L.J., Sercombe T.B., Dilley R.J., Doyle B.J. (2018). Mechanical behaviour of alginate-gelatin hydrogels for 3D bioprinting. J. Mech. Behav. Biomed. Mater..

[5] Cattelan G., Guerrero Gerbolés A., Foresti R., Pramstaller P.P., Rossini A., Miragoli M., Caffarra Malvezzi C. (2020). Alginate Formulations: Current Developments in the Race for Hydrogel-Based Cardiac Regeneration. Front. Bioeng. Biotechnol..

[6] Zhang Y., Li HXu H., Wang L., Zhang M., Liu J., Tan F. (2021). Alginate hydrogels crosslinked with different strontium-calcium ratios as injectable scaffolds for bone tissue engineering. J. Mater. Sci..

[7] Rosińska K., Bartniak M., Wierzbicka A., Sobczyk-Guzenda A., Bociaga D. (2023). Solvent types used for the preparation of hydrogels determine their mechanical properties and influence cell viability through gelatine and calcium ions release. J. Biomed. Mater. Res. B Appl. Biomater..

[8] Neves M.I., Moroni L., Barrias C.C. (2020). Modulating Alginate Hydrogels for Improved Biological Performance as Cellular 3D Microenvironments. Front. Bioeng. Biotechnol..

[9] Sawyer S.W., Takeda K., Alayoubi A., Mirdamadi E., Zidan A., Bauer S.R., Degheidy H. (2023). 3D bioprinting optimization of human mesenchymal stromal cell laden gelatin-alginate-collagen bioink. Biomed. Mater..

[10] Gorgieva S., Kokol V., Pignatello R. (2011). Collagen-vs. Gelatine-Based Biomaterials and Their Biocompatibility: Review and Perspectives. Biomaterials Applications for Nanomedicine.

[11] Wang X., Ao Q., Tian X., Fan J., Tong H., Hou W., Bai S. (2017). Gelatin-based hydrogels for organ 3D bioprinting. Polymers.

[12] Mousavi S., Khoshfetrat A.B., Khatami N., Ahmadian M., Rahbarghazi R. (2019). Comparative study of collagen and gelatin in chitosan-based hydrogels for effective wound dressing: Physical properties and fibroblastic cell behavior. Biochem. Biophys. Res. Commun..

[13] Sarker B., Papageorgiou D.G., Silva R., Zehnder T., Gul-E-Noor F., Bertmer M., Kaschta J., Chrissafis K., Detsch R., Boccaccini A.R. (2014). Fabrication of alginate-gelatin crosslinked hydrogel microcapsules and evaluation of the microstructure and physico-chemical properties. J. Mater. Chem. B.

[14] Waszkiewicz-Robak B., Swiderski F. (2001). Hydrokoloidy pochodzenia roślinnego jako zamienniki żelatyny. Bezpieczna Żywność.

[15] Thakur G., Mitra A., Basak A. (2011). Genipin crosslinked drug-gelatin composite for drug transport and cytocompatibility. Biomed. Eng..

[16] Tonda-Turo C., Gentile P., Saracino S., Chiono V., Nandagiri V.K., Muzio G., Canuto R.A., Ciardelli G. (2011). Comparative analysis of gelatin scaffolds crosslinked by genipin and silane coupling agent. Int. J. Biol. Macromol..

[17] Sarrigiannidis S.O., Rey J.M., Dobre O., González-García C., Dalby M.J., Salmeron-Sanchez M. (2021). A tough act to follow: Collagen hydrogel modifications to improve mechanical and growth factor loading capabilities. Mater. Today Bio.

[18] Stepanovska J., Supova M., Hanzalek K., Broz A., Matejka R. (2021). Collagen bioinks for bioprinting: A systematic review of hydrogel properties, bioprinting parameters, protocols, and bioprinted structure characteristics. Biomedicines.

[19] Hu T., Lo A.C.Y. (2021). Collagen–alginate composite hydrogel: Application in tissue engineering and biomedical sciences. Polymers.

[20] Subhan F., Hussain Z., Tauseef I., Shehzad A., Wahid F. (2021). A review on recent advances and applications of fish collagen. Crit. Rev. Food Sci. Nutr..

[21] Yamada S., Yamamoto K., Ikeda T., Yanagiguchi K., Hayashi Y. (2014). Potency of fish collagen as a scaffold for regenerative medicine. Biomed. Res. Int..

[22] Yang X., Lu Z., Wu H., Li W., Zheng L., Zhao J. (2018). Collagen-alginate as bioink for three-dimensional (3D) cell printing based cartilage tissue engineering. Mater. Sci. Eng. C.

[23] Sarker B., Singh R., Silva R., Roether J.A., Kaschta J., Detsch R., Schubert D.W., Cicha I., Boccaccini A.R. (2014). Evaluation of fibroblasts adhesion and proliferation on alginate-gelatin crosslinked hydrogel. PLoS ONE.

[24] Panwar A., Tan L.P. (2016). Current status of bioinks for micro-extrusion-based 3D bioprinting. Molecules.

[25] Jiao T., Lian Q., Lian W., Wang Y., Li D., Reis R.L., Oliveira J.M. (2023). Properties of Collagen/Sodium Alginate Hydrogels for Bioprinting of Skin Models. J. Bionic Eng..

[26] Karami A., Tebyanian H., Sayyad Soufdoost R., Motavallian E., Barkhordari A., Nourani M.R. (2019). Extraction and Characterization of Collagen with Cost-Effective Method from Human Placenta for Biomedical Applications. World J. Plast. Surg..

[27] Matinong AM E., Chisti Y., Pickering K.L., Haverkamp R.G. (2022). Collagen Extraction from Animal Skin. Biology.

[28] Kittiphattanabawon P., Benjakul S., Visessanguan W., Kishimura H., Shahidi F. (2010). Isolation and Characterisation of collagen from the skin of brownbanded bamboo shark (*Chiloscyllium punctatum*). Food Chem..

[29] Veeruraj A., Arumugam M., Balasubramanian T. (2013). Isolation and characterization of thermostable collagen from the marine eel-fish (*Evenchelys macrura*). Process Biochem..

[30] Maher M., Glattauer V., Onofrillo C., Duchi S., Yue Z., Hughes T.C., Ramshaw JA M., Wallace G.G. (2022). Suitability of Marine-and Porcine-Derived Collagen Type I Hydrogels for Bioprinting and Tissue Engineering Scaffolds. Mar. Drugs.

[31] Coppola D., Oliviero M., Vitale G.A., Lauritano C., D’Ambra I., Iannace S., de Pascale D. (2020). Marine collagen from alternative and sustainable sources: Extraction, processing and applications. Mar. Drugs.

[32] Benjakul S., Nalinanon S., Shahidi F. (2012). Fish Collagen. Food Biochemistry and Food Processing.

[33] Jafari H., Lista A., Siekapen M.M., Ghaffari-Bohlouli P., Nie L., Alimoradi H., Shavandi A. (2020). Fish collagen: Extraction, characterization, and applications for biomaterials engineering. Polymers.

[34] Furtado M., Chen L., Chen Z., Chen A., Cui W. (2022). Development of fish collagen in tissue regeneration and drug delivery. Eng. Regen..

[35] Hackett C., Grim B.J. (2012). The Global Religious Landscape A Report on the Size and Distribution of the World’s Major Religious Groups as of 2010.

[36] Tang Y., Jin S., Li X., Li X., Hu X., Chen Y., Huang F., Yang Z., Yu F., Ding G. (2018). Physicochemical properties and biocompatibility evaluation of collagen from the skin of giant croaker (*Nibea japonica*). Mar. Drugs.

[37] Yu F., Zong C., Jin S., Zheng J., Chen N., Huang J., Chen Y., Huang F., Yang Z., Tang Y. (2018). Optimization of extraction conditions and characterization of pepsin-solubilised collagen from skin of giant croaker (*Nibea japonica*). Mar. Drugs.

[38] Yang F., Jin S., Tang Y. (2019). Marine collagen peptides promote cell proliferation of NIH-3T3 fibroblasts via NF-κB signaling pathway. Molecules.

[39] Zhang K., Fu Q., Yoo J., Chen X., Chandra P., Mo X., Song L., Atala A., Zhao W. (2017). 3D bioprinting of urethra with PCL/PLCL blend and dual autologous cells in fibrin hydrogel: An in vitro evaluation of biomimetic mechanical property and cell growth environment. Acta Biomater..

[40] Xu K., Han Y., Huang Y., Wei P., Yin J., Jiang J. (2022). The application of 3D bioprinting in urological diseases. Mater. Today Bio.

[41] Zhao Y., Liu Y., Dai Y., Yang L., Chen G. (2022). Application of 3D Bioprinting in Urology. Micromachines.

[42] Zhang Y.S., Yue K., Aleman J., Mollazadeh-Moghaddam K., Bakht S.M., Yang J., Jia W., Dell’Erba V., Assawes P., Shin S.R. (2017). 3D Bioprinting for Tissue and Organ Fabrication. Ann. Biomed. Eng..

[43] Das S., Basu B. (2019). An Overview of Hydrogel-Based Bioinks for 3D Bioprinting of Soft Tissues. J. Indian. Inst. Sci..

[44] Bejoy A.M., Makkithaya K.N., Hunakunti B.B., Hegde A., Krishnamurthy K., Sarkar A., Lobo C.F., Keshav D.V.S., Dharshini G., Dhivya Dharshini S. (2021). An insight on advances and applications of 3d bioprinting: A review. Bioprinting.

[45] Wierzbicka A., Bartniak M., Rosińska K., Bociąga D. (2022). Optimization of the Preparation Process Stages of the Bioink Compositions Based on Sodium Alginate and Gelatin to Improve the Viability of Biological Material Contained in Hydrogel 3D Printouts. Eng. Biomater..

[46] Sionkowska A., Musiał K., Gadomska M., Adamiak K. (2022). Fish Collagen and Chitosan Mixtures as a Promising Biomaterial for Potential Use in Medicine and Cosmetic Industry. Eng. Biomater..

[47] Montalbano G., Toumpaniari S., Popov A., Duan P., Chen J., Dalgarno K., Scott W.E., Ferreira A.M. (2018). Synthesis of bioinspired collagen/alginate/fibrin based hydrogels for soft tissue engineering. Mater. Sci. Eng. C.

[48] Ratanavaraporn J., Damrongsakkul S., Sanchavanakit N., Banaprasert T., Kanokpanont S. (2006). Comparison of Gelatin and Collagen Scaffolds for Fibroblast Cell Culture. J. Met. Mater. Miner..

[49] Li Y., Tanaka T. (1990). Kinetics of swelling and shrinking of gels. J. Chem. Phys..

[50] Mondal A., Gebeyehu A., Miranda M., Bahadur D., Patel N., Ramakrishnan S., Rishi A.K., Singh M. (2019). Characterization and printability of Sodium alginate -Gelatin hydrogel for bioprinting NSCLC co-culture. Sci. Rep..

[51] Osidak E.O., Karalkin P.A., Osidak M.S., Parfenov V.A., Sivogrivov D.E., Pereira F.D.A.S., Gryadunova A.A., Koudan E.V., Khesuani Y.D., Kasyanov V.A. (2019). Viscoll collagen solution as a novel bioink for direct 3D bioprinting. J. Mater. Sci. Mater. Med..

[52] Evingür G.A., Sağlam N.A., Çimen B., Uysal B.Ö., Pekcan Ö. (2021). The WS2 dependence on the elasticity and optical band gap energies of swollen PAAm composites. J. Compos. Mater..

[53] Łabowska M.B., Cierluk K., Jankowska A.M., Kulbacka J., Detyna J., Michalak I. (2021). A review on the adaption of alginate-gelatin hydrogels for 3D cultures and bioprinting. Materials.

[54] Lee K.Y., Mooney D.J. (2012). Alginate: Properties and biomedical applications. Prog. Polym. Sci..

[55] Shoichet M.S., Li R.H., White M.L., Winn S.R. (1996). Stability of hydrogels used in cell encapsulation: An in vitro comparison of alginate and agarose. Biotechnol. Bioeng..

[56] Serafin A., Culebras M., Collins M.N. (2023). Synthesis and evaluation of alginate, gelatin, and hyaluronic acid hybrid hydrogels for tissue engineering applications. Int. J. Biol. Macromol..

[57] Chatterjee S., Bohidar H.B. (2005). Effect of cationic size on gelation temperature and properties of gelatin hydrogels. Int. J. Biol. Macromol..

[58] Cranck J. (1956). Diffusion with Moving Boundary. The Mathematics of Diffusion.

[59] Chen Y., Zhou Y., Wang C. (2022). Investigation of Collagen-Incorporated Sodium Alginate Bioprinting Hydrogel for Tissue Engineering. J. Compos. Sci..

[60] Geevarghese R., Somasekharan L.T., Bhatt A., Kasoju N., Nair R.P. (2022). Development and evaluation of a multicomponent bioink consisting of alginate, gelatin, diethylaminoethyl cellulose and collagen peptide for 3D bioprinting of tissue construct for drug screening application. Int. J. Biol. Macromol..

[61] BioRender.com. https://www.biorender.com/.

[62] (2009). European Standard EN ISO 10993-5 Biological Evaluation of Medical Devices—Part 5: Tests for In Vitro Cytotoxicity.

